# Brain correlates of speech perception in schizophrenia patients with and without auditory hallucinations

**DOI:** 10.1371/journal.pone.0276975

**Published:** 2022-12-16

**Authors:** Joan Soler-Vidal, Paola Fuentes-Claramonte, Pilar Salgado-Pineda, Nuria Ramiro, María Ángeles García-León, María Llanos Torres, Antonio Arévalo, Amalia Guerrero-Pedraza, Josep Munuera, Salvador Sarró, Raymond Salvador, Wolfram Hinzen, Peter McKenna, Edith Pomarol-Clotet

**Affiliations:** 1 FIDMAG Hermanas Hospitalarias Research Foundation, Sant Boi de Llobregat, Spain; 2 CIBERSAM (G15), Barcelona, Spain; 3 Universitat de Barcelona, Barcelona, Spain; 4 Benito Menni Complex Asistencial en Salut Mental, Sant Boi de Llobregat, Spain; 5 Hospital de Sant Rafael, Barcelona, Spain; 6 Hospital Mare de Déu de la Mercè, Barcelona, Spain; 7 Hospital Sagrat Cor de Martorell, Barcelona, Spain; 8 Diagnostic Imaging and Image Guided Therapy, Institut de Recerca Sant Joan de Déu, Santa Rosa 39–57, Esplugues de Llobregat, Spain; 9 Diagnostic Imaging Department, Hospital Sant Joan de Déu, Passeig Sant Joan de Déu 2, Barcelona, Spain; 10 Hospital de Sant Joan de Déu, Barcelona, Spain; 11 Universitat Pompeu Fabra, Barcelona, Spain; 12 Institució Catalana de Recerca i Estudis Avançats (ICREA), Barcelona, Spain; Nanyang Technological University, SINGAPORE

## Abstract

The experience of auditory verbal hallucinations (AVH, “hearing voices”) in schizophrenia has been found to be associated with reduced auditory cortex activation during perception of real auditory stimuli like tones and speech. We re-examined this finding using 46 patients with schizophrenia (23 with frequent AVH and 23 hallucination-free), who underwent fMRI scanning while they heard words, sentences and reversed speech. Twenty-five matched healthy controls were also examined. Perception of words, sentences and reversed speech all elicited activation of the bilateral superior temporal cortex, the inferior and lateral prefrontal cortex, the inferior parietal cortex and the supplementary motor area in the patients and the healthy controls. During the sentence and reversed speech conditions, the schizophrenia patients as a group showed reduced activation in the left primary auditory cortex (Heschl’s gyrus) relative to the healthy controls. No differences were found between the patients with and without hallucinations in any condition. This study therefore fails to support previous findings that experience of AVH attenuates speech-perception-related brain activations in the auditory cortex. At the same time, it suggests that schizophrenia patients, regardless of presence of AVH, show reduced activation in the primary auditory cortex during speech perception, a finding which could reflect an early information processing deficit in the disorder.

## Introduction

Auditory verbal hallucinations (AVH) are a core, often distressing, symptom of schizophrenia which are estimated to occur in around 70% of patients [1]. Their clinical features are well established: they may be single or multiple, are often derogatory but less commonly neutral or praising, and they can be experienced as originating inside and outside the head (or both) [1, 2]. Nevertheless, despite research stretching back over more than half a century [3, 4], their underlying basis or bases remain uncertain.

Theoretical approaches to AVH can be classified into two broad categories. One, often termed the ‘neurological’ model, implicates primarily perceptual mechanisms [5–8]. This approach can be traced back to Kraepelin [9] who proposed that AVH were due to pathological (‘irritative’) neuronal activity in the auditory cortex. Modern versions of the theory, however, tend to be more complicated, proposing not only ‘bottom-up’ perceptual mechanisms, but also ‘top-down’ cognitive influences which act to give AVH their specific characteristics, such as being the voices of family, friends, or people engaged in supposed conspiracies against the patient [8]. The other, ‘cognitive’ model, maintains that AVH are the result of cognitive activity that is for unknown reasons misinterpreted as perceptual, for example, inner speech that fails to be labeled as such, or vivid, intrusive memories [10, 11] (for a review see Jones [6]).

Functional imaging has played a key role in the investigation of AVH. Several studies have employed the so-called symptom capture paradigm, which compares brain activity when patients hear a voice (which they signal by a button press) to periods where they do not experience them. Some of these studies, in line with the perceptual model, have found AVH-related activation in the superior temporal cortex, which contains the primary auditory cortex (Heschl’s gyrus) [12–14]. Others, however, have found little or no evidence of temporal cortical activation [15–17].

If the mechanisms underlying AVH involve aberrant perceptual activity, it might also be expected that experiencing them will have consequences for brain activity in response to real sounds or speech that are perceived at the same time. While the simultaneous experience of AVH and real auditory stimuli could in principle lead to either greater than normal activation in the auditory cortex (due to summation of activations), or to reduced activation (due to competition for processing resources), in practice the latter has invariably been assumed [8]. This proposal has so far been examined in three studies. David et al. [18] found attenuation to the point of near extinction of activations to real speech in the auditory cortex in a single case study of a continuously hallucinating patient; when his hallucinations improved, activation to real speech sounds increased. Later, the same group [19] examined 8 patients with schizophrenia with a history of hallucinations and 7 without such a history while they listened to external speech. Relative to 8 healthy comparison subjects, both patient groups showed reduced activation in the left superior temporal gyrus and the auditory association cortex. In 7 patients whose hallucinations subsequently improved, activation in the left superior temporal gyrus and the right middle temporal gyrus increased. Plaze et al. [20] examined 15 patients with daily AVH who underwent fMRI while listening to spoken sentences. Whole brain correlational analysis revealed an inverse association between scores on one of two rating scales for AVH in the posterior part of the left superior temporal gyrus, and a similar inverse association was found on the other scale using region of interest (ROI) analysis.

The aim of the present study was to further examine, using fMRI and whole-brain, voxel-based analysis, whether experience of AVH in schizophrenia is associated with changed auditory cortex responses to perception of real speech. We examined groups of patients with schizophrenia with and without current AVH, also requiring that the frequency was high in the former group. We employed a speech perception paradigm incorporating three different conditions, hearing words, sentences, and reversed speech, to determine whether any potential effects on auditory cortex activations might be related to phonetic-acoustic properties of speech, its lexical structure, or presence of sentential meaning. We additionally examined the findings in the whole group of schizophrenia patients compared to matched healthy controls.

## Results

The final sample consisted of 23 AVH+ and 23 AVH- patients (from initial samples of 29 and 36, respectively, see Methods), and 25 healthy controls. As shown in Table 1, the three groups were matched for age, sex and estimated premorbid IQ, and the two patient groups did not differ significantly in current IQ. In the AVH+ group, hallucination frequency, as measured over a 5-minute period (see Methods), ranged from 0 to 101 instances (mean = 16.19, SD = 22.54, median = 8).

**Table 1:** Demographic and clinical data from the final samples.

	HC	SCHZ	AVH+	AVH-	Differences
	N = 25	N = 46	N = 23	N = 23	
Age	39.8 (14.08)	42.52 (10.72)	40.09 (12.97)	44.96 (7.38)	HC vs SCZ: t = -0.84, p = 0.404 AVH+ vs AVH-: t = -1.57, p = 0.127
Sex (M:F)	18:7	36:10	20:3	16:7	HC vs SCZ: χ^2^ = 0.35, p = 0.555 AVH+ vs AVH-: χ^2^ = 2.04, p = 0.284
Estimated pre-morbid IQ (TAP)	101.17 (9.91)	99.82 (9.27)	98.39 (9.61)	101.32 (8.89)	HC VS SCZ: t = 0.55, p = 0.586 AVH+ vs AVH-: t = -1.06, p = 0.294
Current IQ (WAIS III)	108.00 (18.71)	95.44 (12.96)	92.09 (13.76)	99.32 (11.08)	HC VS SCZ: t = 2.76, p = 0.010 AVH+ vs AVH-: t = -1.83, p = 0.072
Duration of illness (years)	-	17.9 (10.40)	14.76 (10.57)	21.37 (9.26)	t = -2.11, p = 0.042
Antipsychotic dose (mg/day, CPZ-Eq)	-	653.06 (807.76)	549.81 (272.01)	756.31 (1114.12)	t = -0.83 p = 0.418
PANSS Total	-	58.27 (14.87)	64.27 (15.36)	52.52 (12.11)	t = 2.84, p = 0.007
PANSS Positive	-	15.07 (6.33)	18.41 (5.84)	11.87 (5.07)	t = 4.00, p < 0.001
PANSS Negative	-	17.04 (6.09)	18.23 (6.91)	15.91 (5.10)	t = 1.27, p = 0.210
PANSS general psychopathology	-	26.16 (7.23)	27.64 (7.93)	24.74 (6.35)	t = 1.35, p = 0.185
PSYRATS—Hallucination subscale	-	-	24.91 (7.32)	-	-
GAF	-	49.44 (14.45)	45.50 (14.86)	54.00 (12.86)	t = -1.96, p = 0.057
CGI Severity	-	4.15 (1.04)	4.50 (0.86)	3.74 (1.10)	t = 2.45, p = 0.020

HC: Healthy controls, SCHZ: Schizophrenia patients, AVH+: Patients with hallucinations, AVH-: Patients without hallucinations. Values are means (SDs) or frequencies (for sex).

### Imaging results

#### Perception of words (words vs baseline)

**Fig 1:**
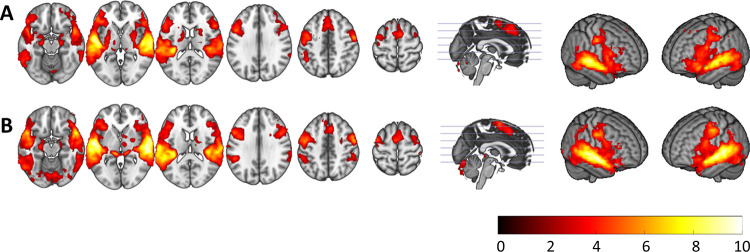
Activation maps for the words condition in healthy controls (A) and patients with schizophrenia (B). Colour bar depicts *Z* values.

In this contrast, the healthy controls showed a pattern of activation involving prominently the temporal lobe cortex, including the bilateral superior and middle temporal cortex (see Fig 1A; S1 Table in S1 File). They also showed activation in the inferior and middle lateral frontal cortex bilaterally, portions of the superior frontal cortex, the insula, pre- and post-central gyri and supplementary motor area. Activation was also seen in the left inferior parietal cortex and the bilateral supramarginal gyri, amygdala, hippocampus/parahippocampus, basal ganglia and thalamus, all bilaterally, and the cerebellum. De-activations were seen in the medial prefrontal cortex, the precuneus and cuneus, and portions of the superior and inferior parietal and posterior temporal cortices, bilaterally. De-activation was also seen the posterior portion of the hippocampus and parahippocampal gyrus and the fusiform and occipital cortex, bilaterally (see S1 Fig and S2 Table in S1 File).

The pattern was broadly similar in the schizophrenia patients. However, in the combined group the de-activations appeared slightly less extensive (see Fig 1B; S1 Fig and S1 Table in S1 File).

Comparison between the healthy controls and the combined group of patients showed no regions of significant differences. There were also no clusters of significant difference between the AVH+ and AVH- patients (see S2 Fig and S1 Table in S1 File for mean activation maps for both patient groups).

#### Perception of sentences (sentences vs baseline)

**Fig 2:**
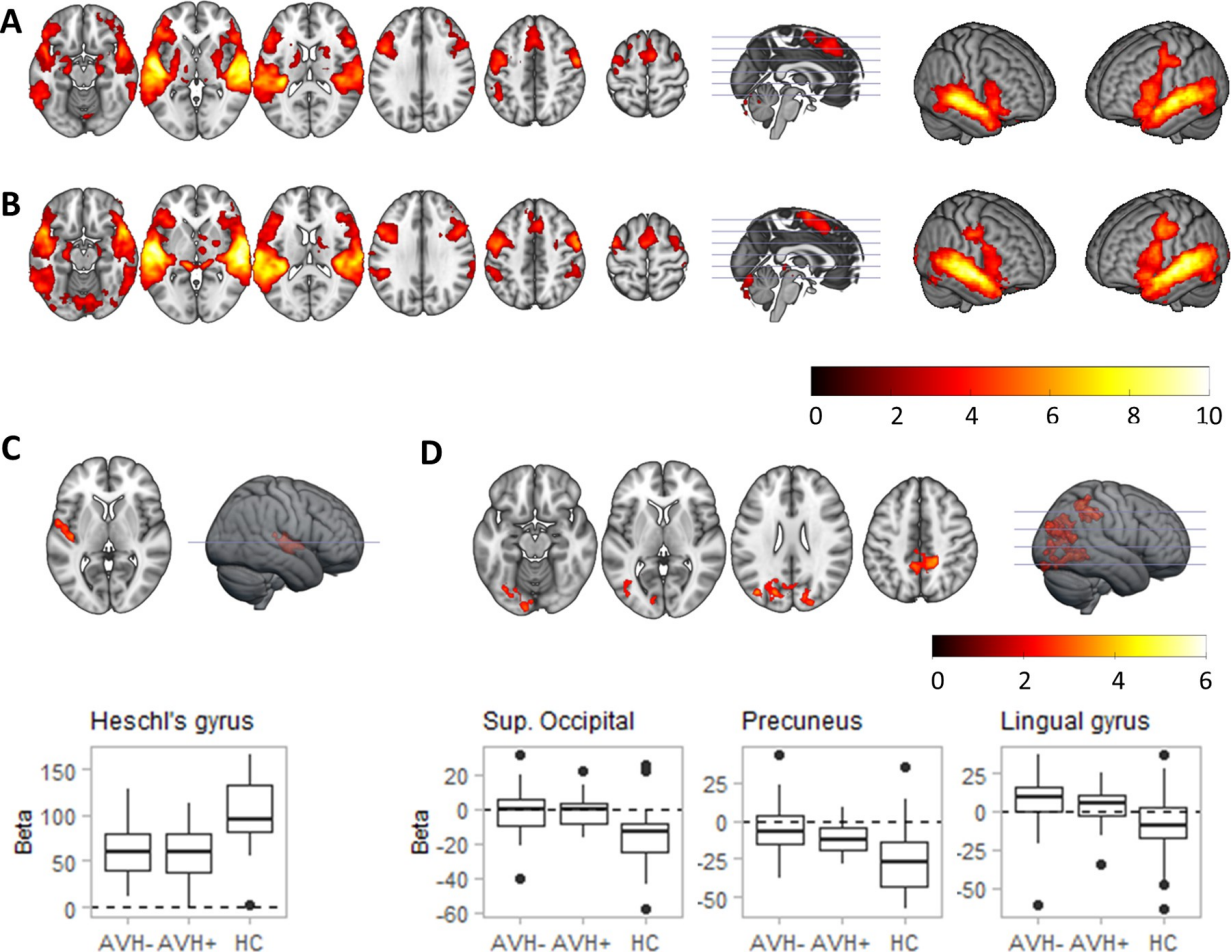
Activation maps for sentences > baseline for healthy controls (A) and patients with schizophrenia (B). (C) and (D) show regions of differences between patients and controls in this contrast, with boxplots depicting parameter estimates (beta values) in each group: (C) shows a region in Heschl’s gyrus hypoactivated in the patients, while regions in (D), corresponding to the superior occipital cortex, the precuneus and the lingual gyrus were hyperactivated in the patients. As shown by the boxplots, this hyperactivation was due to a lack of deactivation in patients. Colour bars depict *Z* values.

In the healthy controls, hearing sentences elicited extensive activation in the superior and middle temporal gyri, the superior and inferior prefrontal cortex, the pre- and post-central gyri and the supplementary motor area, as well as the left angular and bilateral supramarginal gyri. Activation was also seen in the bilateral amygdala, hippocampus/parahippocampus, basal ganglia, thalamus, and cerebellum (Fig 2A; S3 Table in S1 File). De-activations were seen in the anterior cingulate and the lateral and medial superior prefrontal cortex. Additional areas of de-activation were observed in the bilateral precuneus and superior parietal cortex, the parahippocampal gyrus, the fusiform and occipital cortex, and the right caudate and putamen (S4 Table and S1 Fig in S1 File).

The combined group of patients with schizophrenia showed a similar pattern of activation and de-activations, although with visually less extension for de-activations (see Fig 2B; S1 Fig and S3 Table in S1 File). Group comparison revealed a cluster of significantly reduced activation in schizophrenia patients in the left superior temporal cortex (Fig 2C), located in Heschl’s gyrus (MNI coordinates *x* = -56, *y* = -2, *z* = 2; Z = 4.03; cluster size = 374 voxels; *p* = 0.032). There was also relatively increased activation in the patients affecting portions of the occipital cortex (MNI coordinates *x* = -38, *y* = -76, *z* = 26; Z = 3.77; cluster size = 1354 voxels; *p* < 0.001), the precuneus (MNI coordinates *x* = 8, *y* = -48, *z* = 54; Z = 4.01; cluster size = 779 voxels; *p* < 0.001) and the lingual gyrus (MNI coordinates *x* = -26, *y* = -64, *z* = -6; Z = 3.72; cluster size = 503 voxels; *p* = 0.006). As indicated in Fig 2D, this reflected failure of de-activation in the patients. As in the *words* contrast, there were no clusters of significant difference between the AVH+ and AVH- groups.

#### Perception of reversed speech (reversed sentences vs baseline)

**Fig 3:**
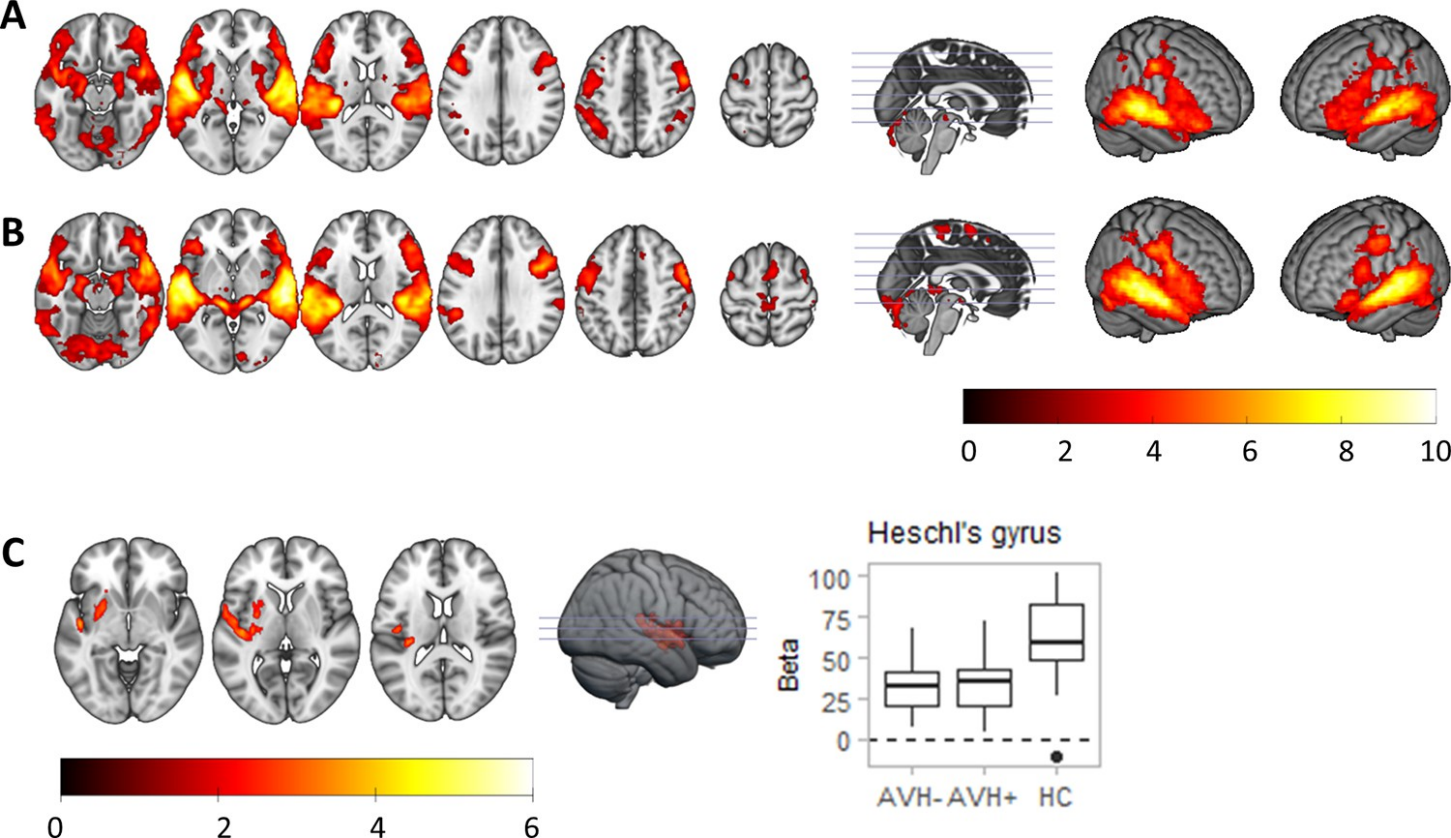
Activation maps for reversed > baseline for healthy controls (A) and patients with schizophrenia (B). (C) shows a region of differences between patients and controls in this contrast, located in Heschl’s gyrus. The boxplot depicts parameter estimates (beta values) in each group, showing hypoactivation in the patients. Color bars depict *Z* values.

The findings here were similar to those in the *sentences* condition. The healthy controls showed activation in the superior, middle, and inferior temporal cortex bilaterally. Activation was also seen in inferior and superior lateral frontal regions and in the pre- and post-central gyri, parts of the inferior and superior parietal cortex, and subcortically in the amygdala, hippocampus and parahippocampus, basal ganglia (on the right only in the caudate) and left thalamus. Regions in the inferior occipital cortex also showed activation (see Fig 3A; S5 Table in S1 File). De-activations were observed in middle and inferior temporal regions bilaterally. The bilateral fusiform gyrus and parts of the hippocampus, parahippocampus, precuneus and cuneus were also de-activated, extending into occipital regions and left superior parietal cortex (S6 Table in S1 File).

The activation and de-activation maps were similar in the combined group of schizophrenia patients (see Fig 3B; S1 Fig and S5 Table in S1 File). Comparison between patients and controls revealed a cluster of reduced activation in the left auditory cortex, involving Heschl’s gyrus and extending into the left putamen (MNI coordinates *x* = -56, *y* = -2, *z* = 2; Z = 4.08; cluster size = 1053 voxels; *p* < 0.001). No group differences were observed for regions of de-activation. As previously, there were no clusters of significant differences between the AVH+ and AVH- patients.

## Discussion

This study found that frequently hallucinating and non-hallucinating schizophrenia patients failed to show activation differences when they heard words, sentences or nonsense (i.e. reversed) speech. Otherwise, we found that, as a group, the patients with schizophrenia showed reduced activation in the left superior temporal cortex in the sentences and reversed sentences conditions, though not when listening to words alone.

Our study fails to replicate a single case study and two group studies [18–20] which previously all found evidence for reduced activation in hallucinating patients during perception of real speech. One further study has also been considered to provide support for this finding. Ford et al. [21] examined a large group of patients with schizophrenia or schizoaffective disorder (N = 109) while they performed auditory oddball task, i.e. they heard one of two tones and had to respond by button press to one of them, which was presented 5% of the time. Compared to 111 healthy controls, the patients showed lower activation in response to the low frequency targets compared to the high frequency tones in four ROIs placed in the auditory cortex (primary auditory cortex, BA41; secondary auditory cortex, BA42; auditory association cortex, BA22; and middle temporal gyrus, BA21). When the patient group was divided into hallucinators (N = 66) and non-hallucinators (N = 40) based on having experienced AVH in the previous week, significantly lower activation was found in the hallucinators in the left primary auditory cortex ROI. Reduced activation in the hallucinators was also seen in bilateral ROIs placed in the visual cortex. Clearly, this study had less than robust findings with respect to AVH, given that reduced activation was only seen in one of four auditory cortex regions examined (and also in a non-auditory region, the visual cortex). It should also be noted that the task used did not measure activations in response to auditory stimuli (tones) *per se*, but rather the difference in activation between commonly and uncommonly presented tones.

Our findings also disagree with a meta-analysis of 11 PET and fMRI studies by Kompus et al. [22]. They found that perception of verbal and non-verbal auditory stimuli in hallucinating schizophrenia patients was associated with reduced activation in the left superior temporal gyrus, among several other areas. However, some of the studies included in this meta-analysis compared patients with moderate or high scores on positive symptoms generally, not high and low scores on AVH specifically. Additionally, activations in the hallucinating/highly symptomatic patients were compared with activations in healthy controls, not non-hallucinating patients, leaving open the possibility that the reduced activation to auditory stimuli found was a function of having schizophrenia generally, not the symptom of AVH specifically.

We in fact found evidence pointing to this latter possibility in our study. The combined group of patients with schizophrenia showed reduced activation in the left primary auditory cortex in two of the three conditions employed, sentences and reversed speech. Although reduced activation in response to a variety of tasks is a common finding in schizophrenia, for example during cognitive control tasks [23], reward anticipation and delivery [24] and emotion processing [25], whether this finding extends to basic auditory perception is uncertain–there have been few relevant studies, and their findings have been conflicting. For example, Woodruff et al. [19] found reduced activation in the left superior temporal gyrus and the auditory association cortex during perception of external speech both schizophrenia patients with and without hallucinations. In a study employing nonverbal auditory stimuli (laughing and crying), Kang et al. [26] found that both 14 hallucinators and 14 non-hallucinators showed areas of reduced activation compared to 28 healthy controls; however, the sites where this was seen did not include the auditory cortex. Braus et al. [27] simultaneously presented visual (a checkerboard) and auditory (drumbeats) stimuli to 12 first episode patients and 11 healthy controls. The patients showed reduced activation in the right thalamus, the right prefrontal cortex, and regions of the parietal lobe, though not the auditory cortex, bilaterally.

We found that the patients with schizophrenia as a group showed failure of de-activation in one of the three conditions used, sentences vs baseline; this was seen in the precuneus, the left superior occipital cortex and the left lingual gyrus. Failure of de-activation is a well-established finding in schizophrenia, and has been found in association with a variety of tasks (for a review see Hu et al. [28]). It is widely considered to reflect dysfunction of the default mode network [29, 30], a set of brain regions, including the medial frontal cortex, the posterior cingulate cortex/precuneus and the angular gyrus, that are normally active at rest but which de-activate during performance of a wide range of attention-demanding tasks. While failure of de-activation in schizophrenia has been most commonly been found to affect the medial frontal cortex, it has also been found in the posterior cingulate cortex/precuneus in some studies [31, 32]. What distinguishes the sentence condition is presence of sentential meaning, i.e., the fact that thoughts are expressed. One possible speculation here is that failure to de-activate in this condition is due to similar mental processes being involved in the brain’s default mode, and schizophrenia involves a failure to segregate between the two networks.

While we found no activation differences between schizophrenia patients with and without AVH using a speech perception task, studies using another auditory perception-related task, mismatch negativity (MMN) have found some evidence of brain functional changes related to this symptom. MMN refers to a wave of negativity that occurs when a sequence of regular auditory stimuli is interrupted by a tone that differs from the remaining along one or more dimensions, for example pitch or duration. MMN amplitude is known to be reduced in patients with schizophrenia [33] and Fisher and co-workers found that indices of MMN attenuation were correlated with hallucination scores [34, 35], and to a trait measure of hallucination proneness [36]. However, numbers were small in these studies (N = 10–12), and the association was not found in another study by the same group [37].

In conclusion, we found no evidence to support the view that presence of auditory hallucinations in patients with schizophrenia reduces activations to real speech, by a presumptive mechanism involving competition for processing resources. A limitation to this negative finding needs to be noted, in that our experimental design was not fine-grained enough to permit measurement of activation to words, sentences and reversed speech at times when AVH were experienced simultaneously with them. A related limitation was that there was considerable variation in the frequency of hallucinations in the AVH+ group, with rates ranging from 0 to over 100 over a five-minute period. It should also be noted that we only examined activations in response to speech stimuli, and it is possible that activations might be different in response to other auditory stimuli such as tones. Despite the negative findings for AVH, we found that the schizophrenia patients as a group showed reduced activation in the left primary auditory cortex during some versions of the auditory perception task. Interpretation of this latter finding is also limited by the above considerations and also by the fact that it emerged unexpectedly, rather than the study being specifically designed to address this possibility. Nevertheless, if genuine, it could possibly point to the presence of an early information processing deficit in the disorder, as argued for by Javitt [38], based on behavioural (e.g. tone matching) and neurophysiological (e.g. event related potential) studies.

## Methods

### Participants

Sixty-five patients meeting DSM-5 criteria for schizophrenia or schizoaffective disorder were initially recruited from four psychiatric hospitals in Barcelona (Benito Menni CASM, Hospital Sagrat Cor de Martorell, Sant Rafael Hospital, Hospital de la Mercè). They were selected based on either experiencing frequent hallucinations (as defined below) (AVH+, N = 29), or having been hallucination-free for at least 6 months (AVH-, N = 36). Diagnoses were made using the Structured Clinical Interview for DSM Disorders (SCID) [39]. Premorbid IQ was estimated using the Word Accentuation Test (Test de Acentuación de Palabras, TAP) [40, 41]. This test requires pronunciation of low-frequency Spanish words whose accents have been removed and is conceptually similar to the English-language National Adult Reading Test (NART) [42] and the Wide Ranging Achievement Test (WRAT) [43]. Current IQ was measured using 4 subtests of the WAIS III (Vocabulary, Similarities, Matrix reasoning and Block design).

Patients were excluded if they (a) were younger than 18 or older than 65, (b) had a history of brain trauma or neurological disease or (c) had shown alcohol/substance abuse/dependence within 12 months prior to participation. Social use of alcohol was permitted, as was non-habitual use of cannabis. Electroconvulsive therapy in the past 6 months was also an exclusion criterion. All participants were right-handed and were taking antipsychotic medication. Based on reasons including failure to complete scanning, excessive head motion, IQ < 70, not being able to recall the task characteristics after scanning, not being completely hallucination-free in the AVH- group, and matching considerations, 23 AVH+ and 23 AVH- patients were finally included.

The control group consisted of 25 healthy individuals, selected to be matched to the two patient groups for age sex and estimated premorbid IQ. They were recruited from non-clinical staff working in the hospitals, their relatives and acquaintances, plus independent sources in the community. They met the same exclusion criteria as the patients, and they were also interviewed using the SCID to exclude current and past psychiatric disorders. They were questioned and excluded if they reported a history of treatment with psychotropic medication beyond non-habitual use of night sedation. Controls were also excluded if they reported a history of major psychiatric disorder in a first-degree relative.

All participants gave written informed consent prior to participation. All the study procedures had been previously approved by the Research Ethics Committee FIDMAG Sisters Hospitallers (Comité de Ética de la Investigación de FIDMAG Hermanas Hospitalarias) and complied with its ethical standards on human experimentation and with the Helsinki Declaration of 1975, as revised in 2008. Healthy controls received a gift-card as a compensation for their participation in the study.

### Clinical and cognitive assessment

AVH severity was assessed with the Psychotic Symptom Rating Scale (PSYRATS [44]), auditory hallucinations subscale (PSYRATS-H). This subscale consists of a semi-structured interview with 11 items referring to frequency, duration, controllability, loudness, location; severity and intensity of distress; amount and degree of negative content; beliefs about the origin of voices; and disruption caused by the AVHs. The PANSS [45] was used to rate positive and negative psychotic symptoms. Overall severity of illness was assessed with the Clinical Global Impression [46] and the Global Assessment of Functioning scale (GAF) [47]. All assessments took place within one week of the scanning session.

Patients in the AVH+ group were required to report hearing AVHs at least once a day (score of 2 in item 1, frequency of voices, in the PSYRATS). To obtain a more accurate measurement of hallucination frequency, they were also asked to remain silent in a quiet environment for 5 minutes and tap on the table every time they heard a voice.

### Speech perception task

While in the scanner, participants performed an auditory speech perception task with three conditions of interest: spoken words (*words*), spoken sentences (*sentences*), and spoken unintelligible reversed speech (*reversed*). These auditory stimuli were presented in a block-design fashion, with six blocks per condition presented in random order, each lasting 26 seconds with a 2-seconds inter-block interval. Every three stimulation blocks, a low-level baseline block was presented consisting of white noise, with the same duration as the speech blocks. The session lasted a total of 11 minutes and 22 seconds.

In the *words* condition, stimuli consisted of unrelated neutral word lists (nouns, verbs, and adjectives). In each block, a list of 22 to 24 words was presented. In the *sentences* condition, a list of 8 unrelated sentences with neutral content was presented in each block. Nouns, verbs, and adjectives from the sentence lists were matched with the ones in the word lists in terms of valence, arousal, and frequency of use, according to normative data from Ferré et al. [48], Guasch et al. [49], Hinojosa et al. [50] and in the EsPal database [51]. Stimuli in the reversed condition consisted of 8 acoustically reversed sentences per block. Stimuli were presented through MRI-compatible headphones (VisuaStim Digital, Resonance Technology, Northridge, CA, USA). To maintain visual stimulation constant and similar for all participants, the task was performed with eyes open while looking at a gray screen shown through MRI-compatible goggles (VisuaStim Digital).

Participants were instructed to remain silent and listen carefully to the recordings during the task. To ensure they had been attending to the presented stimuli, a brief questionnaire was administered immediately after scanning about the type of content heard during the task. Participants also reported their level of attention and, in the case of AVH+ patients, the frequency of hallucinations during the task. Participants who reported not attending to the task or were unable to recall the task characteristics (i.e., they reported hearing something different to word lists, sentence lists and reversed speech, or reported not hearing one of these type of stimuli) were excluded from the analyses.

### Image acquisition

Images were acquired with a 3T Philips Ingenia scanner (Philips Medical Systems, Best, The Netherlands). Functional data were acquired using a T2*-weighted echo-planar imaging (EPI) sequence with 341 volumes and the following acquisition parameters: TR = 2000ms, TE = 30ms, flip angle = 70°, in-plane resolution = 3.5 × 3.5mm, FOV = 238 × 245mm, slice thickness = 3.5mm, inter-slice gap = 0.75mm. Slices (32 per volume) were acquired with an interleaved order parallel to the AC-PC plane. We also acquired a high-resolution anatomical volume with an FFE (Fast Field Echo) sequence for anatomical reference and inspection (TR = 9.90ms; TE = 4.60ms; Flip angle = 8°; voxel size = 1 × 1mm; slice thickness = 1mm; slice number = 180; FOV = 240mm).

### Image preprocessing and analysis

Preprocessing and analysis were carried out with the FEAT module included in the FSL (FMRIB Software Library) software [52]. The first 10 seconds (5 volumes) of the sequence, corresponding to signal stabilization, were discarded. Preprocessing included motion correction (using the MCFLIRT algorithm), co-registration and normalization to a common stereotactic space (MNI, Montreal Neurological Institute template). Brain extraction was first applied to the structural image and the functional sequence was registered to it. Then the structural image was registered to the standard template. These two transformations were used to finally register the functional sequence to the standard space. Before group analyses, normalized images were spatially filtered with a Gaussian filter (FWHM = 5mm). To minimize unwanted movement-related effects, individuals with an estimated maximum absolute movement > 3.0mm or an average absolute movement > 0.3mm were excluded from the study.

Statistical analysis was performed by means of a General Linear Model (GLM) approach. At the first level (within-subject) analysis, separate regressors were defined for the *words*, *sentences*, and *reversed* conditions (white noise blocks were not modeled and thus acted as the implicit baseline). Motion parameters obtained from realignment were also included as nuisance covariates. GLMs were fitted to generate individual activation maps for each condition of interest against the white noise baseline. Second level (group) analyses were performed within the FEAT module by means of mixed-effects GLMs [53], to obtain mean activation maps for each group with one-sample t-tests. Two-sample t-tests were performed to compare the patient and control groups, on the one hand, and the two patient subgroups (AVH+ vs. AVH-), on the other. Group analyses were carried out with sex, age, and pre-morbid IQ as nuisance covariates. All statistical tests were carried out at the cluster level with a corrected *p* < 0.05 using Gaussian random field methods, with a cluster-forming threshold of *z* > 2.3. To identify the regions activated by the task or showing differences between groups, we used the MNI coordinates on the AAL atlas (Anatomical Automatic Labeling) [54].

## Supporting information

S1 File (DOCX)

## References

[1] McCarthy-Jones S. Hearing voices: the histories, causes and meanings of auditory verbal hallucinations. Cambridge: Cambridge University Press; 2012.

[2] Nayani TH, David AS. The auditory hallucination: a phenomenological survey. Psychol Med. 1996. doi: 10.1017/s003329170003381x 8643757

[3] Waters F. Multidisciplinary approaches to understanding auditory hallucinations in schizophrenia and nonschizophrenia populations: the International Consortium on Hallucination Research. Schizophr Bull. 2012;38: 693–694. doi: 10.1093/schbul/sbs070 22837351 PMC3406516

[4] Ford JM, Morris SE, Hoffman RE, Sommer I, Waters F, McCarthy-Jones S, et al. Studying hallucinations within the NIMH RDoC framework. Schizophr Bull. 2014;40 Suppl 4: S295–304. doi: 10.1093/schbul/sbu011 24847862 PMC4141312

[5] David AS. The cognitive neuropsychiatry of auditory verbal hallucinations: an overview. Cogn Neuropsychiatry. 2006/03/31. 2004;9: 107–123. 16LLB3G0EDXNUEQH [pii] doi: 10.1080/13546800344000183 16571577

[6] Jones SR. Do we need multiple models of auditory verbal hallucinations? Examining the phenomenological fit of cognitive and neurological models. Schizophr Bull. 2010;36: 566–575. doi: 10.1093/schbul/sbn129 18820262 PMC2879699

[7] Kompus K, Falkenberg LE, Bless JJ, Johnsen E, Kroken RA, Kråkvik B, et al. The role of the primary auditory cortex in the neural mechanism of auditory verbal hallucinations. Front Hum Neurosci. 2013;7: 144. doi: 10.3389/fnhum.2013.00144 23630479 PMC3633947

[8] Hugdahl K. Auditory hallucinations: A review of the ERC “VOICE” project. World J Psychiatry. 2015/06/26. 2015;5: 193–209. doi: 10.5498/wjp.v5.i2.193 26110121 PMC4473491

[9] Kraepelin E. Dementia praecox and paraphrenia (trans. R.M. Barclay, 1919). Edinburgh: Livingstone; 1913.

[10] Frith CD. The Cognitive Neuropsychology of Schizophrenia. London: Psychology Press; 1992.

[11] Waters FA V, Badcock JC, Michie PT, Maybery MT. Auditory hallucinations in schizophrenia: intrusive thoughts and forgotten memories. Cogn Neuropsychiatry. 2006;11: 65–83. doi: 10.1080/13546800444000191 16537234

[12] Shergill SS, Brammer MJ, Williams SCR, Murray RM, McGuire PK. Mapping auditory hallucinations in schizophrenia using functional magnetic resonance imaging. Arch Gen Psychiatry. 2000/11/14. 2000;57: 1033–1038. doi: 10.1001/archpsyc.57.11.1033 11074868

[13] Copolov DL, Seal ML, Maruff P, Ulusoy R, Wong MT, Tochon-Danguy HJ, et al. Cortical activation associated with the experience of auditory hallucinations and perception of human speech in schizophrenia: a PET correlation study. Psychiatry Res. 2003/04/16. 2003;122: 139–152. S092549270200121X [pii] doi: 10.1016/s0925-4927(02)00121-x 12694889

[14] Diederen KMJ, Neggers SFW, Daalman K, Blom JD, Goekoop R, Kahn RS, et al. Deactivation of the parahippocampal gyrus preceding auditory hallucinations in schizophrenia. Am J Psychiatry. 2010/02/04. 2010;167: 427–435. appi.ajp.2009.09040456 [pii] doi: 10.1176/appi.ajp.2009.09040456 20123912

[15] Silbersweig DA, Stern E, Frith C, Cahill C, Holmes A, Grootoonk S, et al. A functional neuroanatomy of hallucinations in schizophrenia. Nature. 1995/11/09. 1995;378: 176–179. doi: 10.1038/378176a0 7477318

[16] Raij TT, Valkonen-Korhonen M, Holi M, Therman S, Lehtonen J, Hari R. Reality of auditory verbal hallucinations. Brain. 2009/07/22. 2009;132: 2994–3001. doi: 10.1093/brain/awp186 19620178 PMC2768657

[17] Sommer IEC, Diederen KMJ, Blom JD, Willems A, Kushan L, Slotema K, et al. Auditory verbal hallucinations predominantly activate the right inferior frontal area. Brain. 2008;131: 3169–3177. doi: 10.1093/brain/awn251 18854323

[18] David AS, Woodruff PW, Howard R, Mellers JD, Brammer M, Bullmore E, et al. Auditory hallucinations inhibit exogenous activation of auditory association cortex. Neuroreport. 1996/03/22. 1996;7: 932–936. doi: 10.1097/00001756-199603220-00021 8724677

[19] Woodruff PW, Wright IC, Bullmore ET, Brammer M, Howard RJ, Williams SC, et al. Auditory hallucinations and the temporal cortical response to speech in schizophrenia: a functional magnetic resonance imaging study. Am J Psychiatry. 1997/12/16. 1997;154: 1676–1682. doi: 10.1176/ajp.154.12.1676 9396945

[20] Plaze M, Bartrés-Faz D, Martinot J-L, Januel D, Bellivier F, De Beaurepaire R, et al. Left superior temporal gyrus activation during sentence perception negatively correlates with auditory hallucination severity in schizophrenia patients. Schizophr Res. 2006;87: 109–115. doi: 10.1016/j.schres.2006.05.005 16828542

[21] Ford JM, Roach BJJ, Jorgensen KW, Turner JA, Brown GGG, Notestine R, et al. Tuning in to the Voices: A Multisite fMRI Study of Auditory Hallucinations. Schizophr Bull. 2008/11/07. 2009;35: 58–66. doi: 10.1093/schbul/sbn140 18987102 PMC2643968

[22] Kompus K, Westerhausen R, Hugdahl K. The “paradoxical” engagement of the primary auditory cortex in patients with auditory verbal hallucinations: A meta-analysis of functional neuroimaging studies. Neuropsychologia. 2011;49: 3361–3369. doi: 10.1016/j.neuropsychologia.2011.08.010 21872614

[23] Minzenberg MJ, Laird AR, Thelen S, Carter CS, Glahn DC. Meta-analysis of 41 functional neuroimaging studies of executive function in schizophrenia. Arch Gen Psychiatry. 2009;66: 811–822. doi: 10.1001/archgenpsychiatry.2009.91 19652121 PMC2888482

[24] Yan C, Yang T, Yu Q jing, Jin Z, Cheung EFC, Liu X, et al. Rostral medial prefrontal dysfunctions and consummatory pleasure in schizophrenia: A meta-analysis of functional imaging studies. Psychiatry Res—Neuroimaging. 2015;231: 187–196. doi: 10.1016/j.pscychresns.2015.01.001 25637357

[25] Li H, Chan RCK, McAlonan GM, Gong QY. Facial emotion processing in schizophrenia: A meta-analysis of functional neuroimaging data. Schizophr Bull. 2010;36: 1029–1039. doi: 10.1093/schbul/sbn190 19336391 PMC2930350

[26] Kang JI, Kim JJ, Seok JH, Chun JW, Lee SK, Park HJ. Abnormal brain response during the auditory emotional processing in schizophrenic patients with chronic auditory hallucinations. Schizophr Res. 2008/09/27. 2009;107: 83–91.S0920-9964(08)00377-0 [pii] doi: 10.1016/j.schres.2008.08.019 18818053

[27] Braus DF, Weber-Fahr W, Tost H, Ruf M, Henn FA. Sensory information processing in neuroleptic-naive first-episode schizophrenic patients: a functional magnetic resonance imaging study. Arch Gen Psychiatry. 2002/08/02. 2002;59: 696–701. yoa10125 [pii] doi: 10.1001/archpsyc.59.8.696 12150645

[28] Hu ML, Zong XF, Mann JJ, Zheng JJ, Liao YH, Li ZC, et al. A Review of the Functional and Anatomical Default Mode Network in Schizophrenia. Neurosci Bull. 2016/12/21. 2017;33: 73–84. doi: 10.1007/s12264-016-0090-1 [pii] 27995564 PMC5567552

[29] Buckner RL, Andrews-hanna JR, Schacter DL. The Brain ‘ s Default Network The Brain ‘ s Default Network Anatomy, Function, and Relevance to Disease. 2008. doi: 10.1196/annals.1440.011 18400922

[30] Gusnard DA, Raichle ME. Searching for a baseline: Functional imaging and the resting human brain. Nat Rev Neurosci. 2001;2: 685–694. doi: 10.1038/35094500 11584306

[31] Salgado-Pineda P, Fakra E, Delaveau P, McKenna PJ, Pomarol-Clotet E, Blin O. Correlated structural and functional brain abnormalities in the default mode network in schizophrenia patients. Schizophr Res. 2011;125: 101–109. doi: 10.1016/j.schres.2010.10.027 21095105

[32] Schneider FC, Royer A, Grosselin A, Pellet J, Barral FG, Laurent B, et al. Modulation of the default mode network is task-dependant in chronic schizophrenia patients. Schizophr Res. 2011;125: 110–117. doi: 10.1016/j.schres.2010.11.013 21147518

[33] Erickson MA, Ruffle A, Gold JM. 乳鼠心肌提取 HHS Public Access. Biol Psychiatry. 2016;79: 980–987. doi: 10.1016/j.biopsych.2015.08.025.A 26444073 PMC4775447

[34] Fisher DJ, Grant B, Smith DM, Borracci G, Labelle A, Knott VJ. Effects of auditory hallucinations on the mismatch negativity (MMN) in schizophrenia as measured by a modified “optimal” multi-feature paradigm. Int J Psychophysiol. 2011;81: 245–251. doi: 10.1016/j.ijpsycho.2011.06.018 21749905

[35] Fisher DJ, Labelle A, Knott VJ. Alterations of mismatch negativity (MMN) in schizophrenia patients with auditory hallucinations experiencing acute exacerbation of illness. Schizophr Res. 2012;139: 237–245. doi: 10.1016/j.schres.2012.06.004 22727705

[36] Fisher DJ, Smith DM, Labelle A, Knott VJ. Attenuation of mismatch negativity (MMN) and novelty P300 in schizophrenia patients with auditory hallucinations experiencing acute exacerbation of illness. Biol Psychol. 2014;100: 43–49. doi: 10.1016/j.biopsycho.2014.05.005 24865523

[37] Fisher DJ, Labelle A, Knott VJ. Auditory hallucinations and the mismatch negativity: Processing speech and non-speech sounds in schizophrenia. Int J Psychophysiol. 2008;70: 3–15. doi: 10.1016/j.ijpsycho.2008.04.001 18511139

[38] Javitt DC. Sensory processing in schizophrenia: neither simple nor intact. Schizophr Bull. 2009/10/17. 2009;35: 1059–1064. sbp110 [pii] doi: 10.1093/schbul/sbp110 19833806 PMC2762632

[39] First MB, Williams JBW, Karg RS, Spitzer RL. Structured clinical interview for DSM-5 research version. Am Psychiatr Assoc Washingt DC. 2015.

[40] Del Ser T, Gonzalez-Montalvo JI, Martinez-Espinosa S, Delgado-Villapalos C, Bermejo F. Estimation of premorbid intelligence in Spanish people with the Word Accentuation Test and its application to the diagnosis of dementia. Brain Cogn. 1997/04/01. 1997;33: 343–356. S0278-2626(97)90877-0 [pii] doi: 10.1006/brcg.1997.0877 9126399

[41] Gomar JJ, Ortiz-Gil J, McKenna PJ, Salvador R, Sans-Sansa B, Sarró S, et al. Validation of the Word Accentuation Test (TAP) as a means of estimating premorbid IQ in Spanish speakers. Schizophr Res. 2011;128: 175–176. doi: 10.1016/j.schres.2010.11.016 21144711

[42] Nelson HE; Willison JR. The Revised National Adult Reading Test. Windsor: NFER-Nelson; 1991.

[43] Wilkinson GS; Robertson GJ. WRAT-5: Wide Range Achievement Test. Professional Manual. 5th ed. Bloomington: Pearson Inc; 2017.

[44] Haddock G, McCarron J, Tarrier N, Faragher EB. Scales to measure dimensions of hallucinations and delusions: The psychotic symptom rating scales (PSYRATS). Psychol Med. 1999. doi: 10.1017/s0033291799008661 10473315

[45] Kay SR, Flszbein A, Opfer LA. The Positive and Negative Syndrome Scale (PANSS) for Schizophrenia. Schizophr Bull. 1987;13: 261–276. doi: 10.1093/schbul/13.2.261 3616518

[46] Guy W. Clinical Global Impression. ECDEU Assessment Manual for Psychopharmacology, revised. Rockville, MD: National Institute of Mental Health; 1976.

[47] American Psychiatric Association. Diagnostic and Statistical Manual of Mental Disorders. American Psychiatric Association; 2013. doi: 10.1176/appi.books.9780890425596

[48] Ferré P, Guasch M, Moldovan C, Sánchez-Casas R. Affective norms for 380 Spanish words belonging to three different semantic categories. Behav Res Methods. 2012;44: 395–403. doi: 10.3758/s13428-011-0165-x 22042646

[49] Guasch M, Ferré P, Fraga I. Spanish norms for affective and lexico-semantic variables for 1,400 words. Behav Res Methods. 2016;48: 1358–1369. doi: 10.3758/s13428-015-0684-y 26542969

[50] Hinojosa JA, Martínez-García N, Villalba-García C, Fernández-Folgueiras U, Sánchez-Carmona A, Pozo MA, et al. Affective norms of 875 Spanish words for five discrete emotional categories and two emotional dimensions. Behav Res Methods. 2015; 1–13. doi: 10.3758/s13428-015-0572-5 25740761

[51] Duchon A, Perea M, Sebastián-Gallés N, Martí A, Carreiras M. EsPal: one-stop shopping for Spanish word properties. Behav Res Methods. 2013;45: 1246–58. doi: 10.3758/s13428-013-0326-1 23468181

[52] Smith SM, Jenkinson M, Woolrich MW, Beckmann CF, Behrens TEJ, Johansen-Berg H, et al. Advances in functional and structural MR image analysis and implementation as FSL. Neuroimage. 2004/10/27. 2004;23 Suppl 1: S208–19. S1053-8119(04)00393-3 [pii] doi: 10.1016/j.neuroimage.2004.07.051 15501092

[53] Beckmann CF, Jenkinson M, Smith SM. General multilevel linear modeling for group analysis in FMRI. Neuroimage. 2003;20: 1052–1063. doi: 10.1016/S1053-8119(03)00435-X 14568475

[54] Tzourio-Mazoyer N, Landeau B, Papathanassiou D, Crivello F, Etard O, Delcroix N, et al. Automated anatomical labeling of activations in SPM using a macroscopic anatomical parcellation of the MNI MRI single-subject brain. Neuroimage. 2002;15: 273–289. doi: 10.1006/nimg.2001.0978 11771995

