# Supplementary material for: Brain correlates of speech perception in schizophrenia patients with and without auditory hallucinations
Source: PLoS One. 2022 Dec 16;17(12):e0276975. doi: 10.1371/journal.pone.0276975 (PMC9757556; doi:10.1371/journal.pone.0276975)
Supplement: S1 File — (DOCX) [file pone.0276975.s001.docx]

**Supporting information**

**Supplementary Figures**

**S1 Figure. Maps for deactivation in the three task conditions for healthy controls (HC) and schizophrenia patients (SCHZ).** Color bar depicts *Z* values.


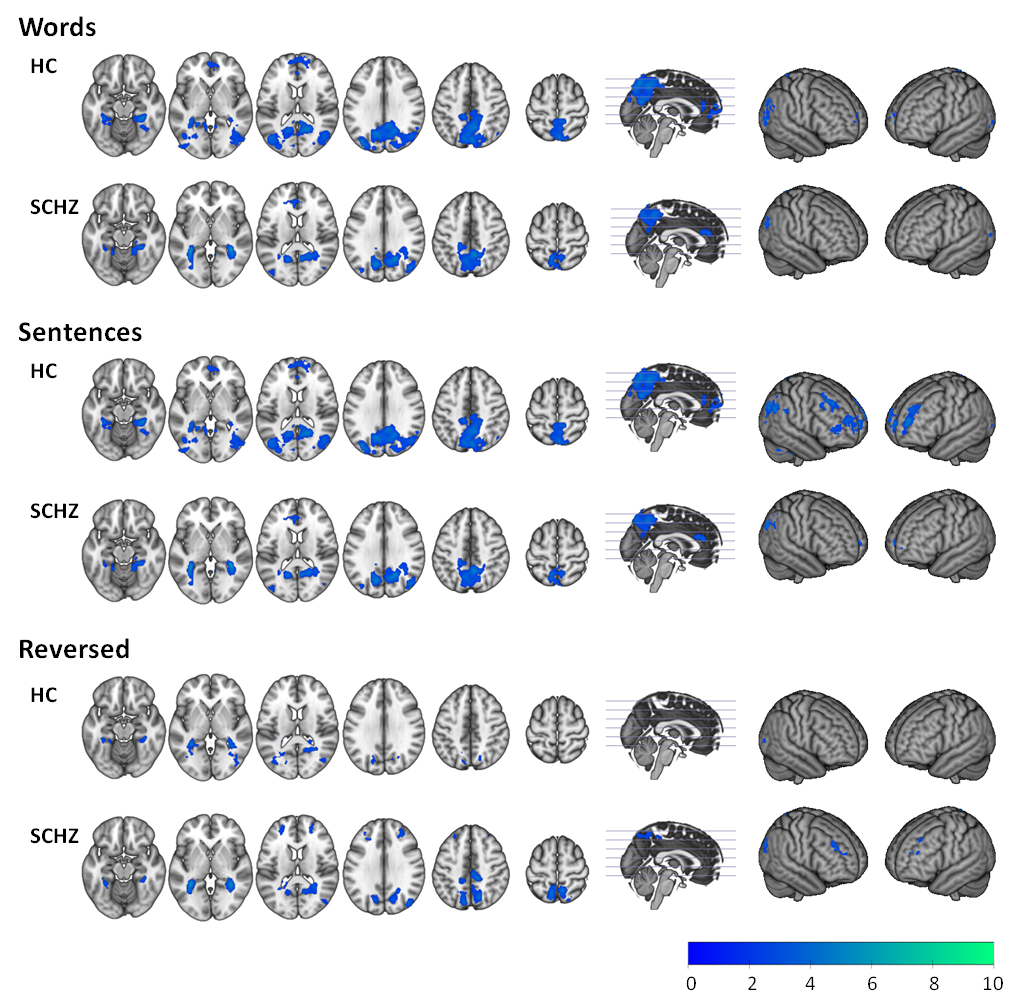


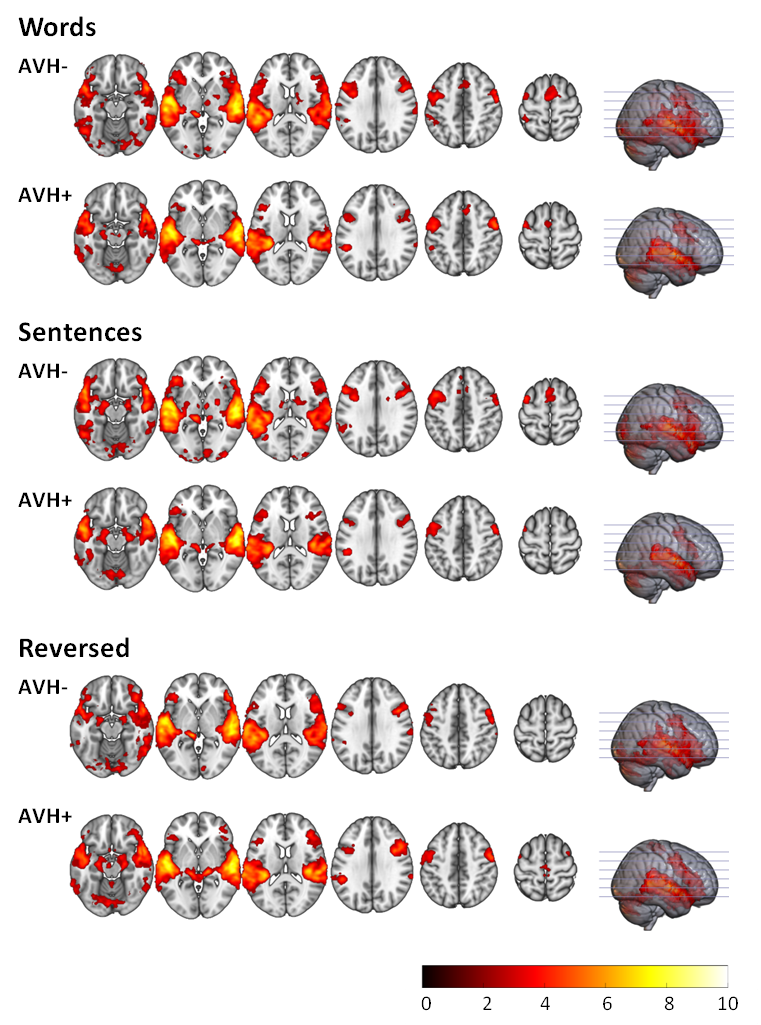
**S2 Figure. Maps for activation in the three task conditions for the AVH- and AVH+ groups separately.** Color bar depicts *Z* values.

**Supplementary Tables**

**S1 Table. Activation during the Words condition (Words > Baseline).** . For large clusters, regions encompassing the area of activation are indicated in brackets.

|  | **MNI coordinates** | | |  |  |  |
| --- | --- | --- | --- | --- | --- | --- |
|  | ***x*** | ***y*** | ***z*** | ***Z*** | ***k*** | ***p*** |
| ***Controls (HC)*** |  |  |  |  |  |  |
|  |  |  |  |  |  |  |
| Superior temporal gyrus (L)  (Pre/post-central gyrus, IFG *triangularis*, IFG *orbitalis*, IFG *opercularis*, supramarginal gyrus, pallidum, putamen, thalamus, middle and inferior temporal gyrus, cerebellum, amygdala, hippocampus) | -52 | -12 | 0 | 9.52 | 20489 | <0.001 |
| Posterior superior temporal gyrus (L) | -34 | -30 | 12 | 8.88 |  |  |
| Superior temporal gyrus (R)  (Pre/post-central gyrus, IFG *triangularis*, IFG *orbitalis*, IFG *opercularis*, supramarginal gyrus, pallidum, putamen, middle and inferior temporal gyrus, amygdala, hippocampus) | 62 | -18 | 2 | 9.47 | 11517 | <0.001 |
| Superior frontal gyrus (L) | -8 | 2 | 52 | 4.73 | 1842 | <0.001 |
| Supplementary motor area (R) | 6 | 2 | 60 | 4.66 |  |  |
| Angular gyrus/Inferior parietal cortex (L) | -46 | -50 | 44 | 4.01 | 379 | 0.0262 |
|  |  |  |  |  |  |  |
| ***Patients (SZ)*** |  |  |  |  |  |  |
| Superior temporal gyrus (R)  (Pre/post-central gyrus, IFG *triangularis*, IFG *orbitalis*, IFG *opercularis*, supramarginal gyrus, pallidum, putamen, thalamus, middle and inferior temporal gyrus, cerebellum, amygdala, hippocampus) | 60 | -26 | 4 | 10.6 | 40792 | <0.001 |
| Superior temporal gyrus (L) | -56 | -12 | 2 | 9.98 |  |  |
| Posterior Superior temporal gyrus (L) | -46 | -26 | 8 | 9.95 |  |  |
| Supplementary motor area (R) | 2 | 0 | 68 | 5.02 | 1722 | <0.001 |
| Superior frontal gyrus (L) | -4 | -4 | 62 | 4.88 |  |  |
| Superior frontal gyrus (R) | 6 | 22 | 50 | 4.76 |  |  |
|  |  |  |  |  |  |  |
| ***AVH-*** |  |  |  |  |  |  |
| Posterior superior temporal gyrus (L)  (Pre/post-central gyrus, IFG *triangularis*, IFG *orbitalis*, IFG *opercularis*, supramarginal gyrus, middle and inferior temporal gyrus, cerebellum, amygdala, hippocampus) | -46 | -26 | 8 | 8.5 | 16553 | <0.001 |
| Superior temporal gyrus (L) | -62 | -24 | 2 | 7.84 |  |  |
| Superior temporal gyrus (R)  (Pre/post-central gyrus, IFG *triangularis*, IFG *orbitalis*, IFG *opercularis*, middle and inferior temporal gyrus, cerebellum) | 60 | -26 | 4 | 9.18 | 11287 | <0.001 |
| Pole of superior temporal gyrus (R) | 58 | 4 | -6 | 8.06 |  |  |
| Superior frontal gyrus (R) | 8 | 10 | 54 | 4.3 | 1054 | <0.001 |
| Superior frontal gyrus (L) | -4 | -4 | 62 | 4.02 |  |  |
| Cerebellum (R) | 26 | -64 | -20 | 4.2 | 1033 | <0.001 |
| Fusiform gyrus (R) | 14 | -88 | -16 | 3.69 |  |  |
| Inferior occipital gyrus (R) | 14 | -86 | -10 | 3.48 |  |  |
| Putamen (R) | 24 | -2 | 8 | 3.39 | 369 | 0.03 |
| Cerebellum (R) | 16 | -78 | -42 | 4.75 | 349 | 0.04 |
|  |  |  |  |  |  |  |
| ***AVH+*** |  |  |  |  |  |  |
| Superior temporal gyrus (L)  (Superior, middle and inferior temporal, supramarginal gyrus, inferior parietal gyrus, hippocampus, amygdala) | -48 | -18 | 2 | 8.26 | 10471 | <0.001 |
| Posterior superior temporal gyrus (L) | -36 | -30 | 10 | 8.1 |  |  |
| Superior temporal gyrus (L)  (Superior, middle and inferior temporal) | -58 | -14 | 2 | 8.57 | 7823 | <0.001 |
| Pole of superior temporal gyrus (R) | 56 | 2 | -6 | 8.1 |  |  |
| Cerebellum (R) | 26 | -62 | -24 | 4.97 | 2156 | <0.001 |
| Cerebellum (L) | -16 | -78 | -38 | 4.11 |  |  |
| Precentral gyrus (L) | -48 | -2 | 40 | 4.25 | 2122 | <0.001 |
| IFG *opercularis* (L) | -40 | 8 | 24 | 4.06 |  |  |
| IFG *triangularis* (L) | -42 | 26 | 14 | 3.72 |  |  |
| Precentral gyrus (R) | 52 | -2 | 44 | 5.54 | 988 | <0.001 |
| Superior frontal gyrus (R) | 6 | 22 | 46 | 3.72 | 674 | <0.001 |

IFG: Inferior frontal gyrus; L: Left; R: Right

**S2 Table. Deactivations in the Words condition (Baseline > Words).**

|  | **MNI coordinates** | | |  |  |  |
| --- | --- | --- | --- | --- | --- | --- |
|  | ***x*** | ***y*** | ***z*** | ***Z*** | ***k*** | ***p*** |
| ***Controls (HC)*** |  |  |  |  |  |  |
| Fusiform gyrus (L)  (Cuneus, middle and posterior occipital) | -34 | -46 | -10 | 5.92 | 14257 | <0.001 |
| Precuneus (R) | 6 | -52 | 52 | 5.44 |  |  |
| Medial superior frontal gyrus (R) | 6 | 60 | 18 | 3.75 | 667 | <0.001 |
| ***Patients (SZ)*** |  |  |  |  |  |  |
| Parahippocampal gyrus (L) | -34 | -42 | -4 | 6.26 | 7763 | <0.001 |
| Precuneus (R) | 24 | -56 | 24 | 5.81 |  |  |
| Cuneus (R) | 8 | -60 | 22 | 5.71 |  |  |
| Middle occipital cortex (R) | 50 | -70 | 26 | 5.03 | 445 | 0.010 |
| Anterior cingulate cortex (L) | -4 | 38 | 18 | 3.99 | 354 | 0.038 |
| ***AVH-*** |  |  |  |  |  |  |
| Precuneus (R) | 22 | -54 | 26 | 4.67 | 2922 | <0.001 |
| Angular gyrus (R) | 38 | -52 | 26 | 4.53 |  |  |
| Cuneus (L) | -16 | -62 | 24 | 4.14 |  |  |
| Posterior thalamic radiation (L) | -34 | -50 | 2 | 4.57 | 630 | <0.001 |
| ***AVH+*** |  |  |  |  |  |  |
| Posterior thalamic radiation (R) | 36 | -42 | 0 | 5.39 | 3850 | <0.001 |
| Superior occipital gyrus (R) | 24 | -56 | 22 | 4.96 |  |  |
| Precuneus (R) | 8 | -62 | 56 | 4.58 |  |  |
| Precuneus (L) | -8 | -56 | 58 | 4.16 |  |  |
| Parahippocampal gyrus (L) | -32 | -42 | -4 | 4.75 | 640 | <0.001 |

L: Left; R: Right

**S3 Table. Activation during the Sentences condition (Sentences > Baseline).** For large clusters, regions encompassing the area of activation are indicated in brackets.

|  | **MNI coordinates** | | |  |  |  |
| --- | --- | --- | --- | --- | --- | --- |
|  | ***x*** | ***y*** | ***z*** | ***Z*** | ***k*** | ***p*** |
| ***Controls (HC)*** |  |  |  |  |  |  |
| Superior temporal gyrus (L)  (Pre/post-central gyrus, IFG *triangularis*, IFG *orbitalis*, IFG *opercularis*, supramarginal gyrus, pallidum, middle and inferior temporal gyrus, amygdala, hippocampus) | -52 | -12 | 9 | 9.69 | 15875 | <0.001 |
| Posterior superior  temporal gyrus (L) | -36 | -30 | 12 | 8.81 |  |  |
| Superior temporal gyrus (R)  (IFG *triangularis*, IFG *orbitalis*, IFG *opercularis*, middle and inferior temporal gyrus) | 48 | -16 | 2 | 9.62 | 8761 | <0.001 |
| Posterior superior temporal gyrus (R) | 52 | -30 | 2 | 8.6 |  |  |
| Cerebellum (R) | 18 | -78 | -42 | 6.86 | 2562 | <0.001 |
| Cerebellum (L) | -16 | -80 | -42 | 6.28 |  |  |
| Hippocampus (R)  (Putamen, pallidum) | 20 | -14 | -16 | 6.31 | 1284 | <0.001 |
| Amygdala (R) | 32 | -2 | -20 | 4.07 |  |  |
| Uncinate Fasciculus (R) | 32 | 2 | -16 | 4.06 |  |  |
| Superior frontal gyrus (L) | -8 | 2 | 58 | 5.24 | 623 |  |
| ***Patients (SZ)*** |  |  |  |  |  |  |
| Superior temporal gyrus (R)  (Pre/post-central gyrus, IFG *triangularis*, IFG *orbitalis*, IFG *opercularis*, supramarginal gyrus, pallidum, middle and inferior temporal gyrus, amygdala, hippocampus) | 48 | -18 | 4 | 10.2 | 41395 | <0.001 |
| Superior temporal gyrus (L) | -56 | -12 | 2 | 9.93 |  |  |
| Inferior frontal gyrus pars triangularis (L) | -44 | 26 | 8 | 9.89 |  |  |
| Supplementary motor area (L) | -12 | 12 | 50 | 4.78 | 952 | <0.001 |
| ***AVH-*** |  |  |  |  |  |  |
| Posterior superior temporal gyrus (L)  (Pre/post-central gyrus, IFG *triangularis*, IFG *orbitalis*, IFG *opercularis*, supramarginal gyrus, pallidum, putamen, middle and inferior temporal gyrus, amygdala, hippocampus) | -48 | -28 | 6 | 8.35 | 18815 | <0.001 |
| Superior temporal gyrus (L) | -58 | -12 | 2 | 8.31 |  |  |
| Superior temporal gyrus (R)  (Pre-central gyrus, IFG *triangularis*, IFG *orbitalis*, IFG *opercularis*, pallidum, putamen, middle and inferior temporal gyrus, amygdala, hippocampus) | 48 | -18 | 4 | 8.54 | 10297 | <0.001 |
| Pole of superior temporal gyrus (R) | 58 | 2 | -6 | 7.68 |  |  |
| Cerebellum/Occipital (R) | 16 | -80 | -42 | 5.84 | 3075 | <0.001 |
| Superior frontal gyrus (R) | 0 | 0 | 66 | 4.24 | 925 | <0.001 |
| ***AVH+*** |  |  |  |  |  |  |
| Superior temporal gyrus (L)  (Pre/post-central gyrus, IFG *triangularis*, IFG *orbitalis*, IFG *opercularis*, supramarginal gyrus, middle and inferior temporal gyrus, amygdala, hippocampus, cerebellum) | -50 | -16 | 0 | 8.2 | 15365 | <0.001 |
| Posterior superior temporal gyrus (L) | -36 | -30 | 10 | 8.12 |  |  |
| Pole of superior temporal gyrus (L) | -54 | 4 | -10 | 7.57 |  |  |
| Superior temporal gyrus (R)  (Middle and inferior temporal gyrus, hippocampus) | 48 | -20 | 2 | 8.34 | 7634 | <0.001 |
| Pole of superior temporal gyrus (R) | 58 | 0 | -6 | 8.01 |  |  |
| Posterior superior temporal gyrus (R) | 52 | -28 | 4 | 7.96 |  |  |
| Precentral gyrus (R) | 52 | -2 | 44 | 4.36 | 1042 | <0.001 |
| IFG *opercularis* (R) | 36 | 18 | 18 | 4.2 |  |  |

IFG: Inferior frontal gyrus; L: Left; R: Right

**S4 Table. Deactivations in the Sentences condition (Baseline > Sentences).**

|  | **MNI coordinates** | | |  |  |  |
| --- | --- | --- | --- | --- | --- | --- |
|  | ***x*** | ***y*** | ***z*** | ***Z*** | ***k*** | ***p*** |
| ***Controls (HC)*** |  |  |  |  |  |  |
| Superior occipital gyrus (L)  (Cuneus, posterior cingulate cortex, fusiform gyrus, supramarginal gyrus, angular gyrus) | -16 | -68 | 40 | 6.4 | 15570 | <0.001 |
| Precuneus (R) | 8 | -48 | 54 | 6.39 |  |  |
| Precuneus (L) | -2 | -50 | 52 | 6.33 |  |  |
| Rostral anterior cingulate gyrus (R) | 0 | 40 | 20 | 5.31 | 6187 | <0.001 |
| Superior frontal cortex (L) | -26 | 58 | 22 | 5.12 |  |  |
| Dorsal anterior cingulate gyrus (L) | -2 | 30 | 26 | 4.87 |  |  |
| Medial superior frontal gyrus (L) | -2 | 62 | 6 | 4.64 |  |  |
| Middle frontal gyrus (L) | -28 | 28 | 36 | 4.6 |  |  |
| Middle frontal gyrus (R) | 26 | 30 | 44 | 5.06 | 1232 | <0.001 |
| Superior frontal gyrus (R) | 20 | 14 | 52 | 4.92 |  |  |
| Cerebellum (R) | 44 | -52 | 40 | 1.89 | 440 | 0.013 |
| ***Patients (SZ)*** |  |  |  |  |  |  |
| Precuneus (L)  (Posterior cingulate cortex, parahippocampal gyrus, fusiform gyrus) | -10 | -66 | 34 | 6.28 | 7435 | <0.001 |
| Precuneus (R) | 16 | -62 | 38 | 5.66 |  |  |
| Anterior cingulate cortex (L) | -2 | 32 | 20 | 4.84 | 1356 | <0.001 |
| Medial superior frontal cortex (R) | 2 | 66 | 10 | 4.10 |  |  |
| Middle occipital cortex (R) | 44 | -74 | 34 | 5.15 | 948 | <0.001 |
| Angular gyrus (R) | 54 | -52 | 30 | 3.80 |  |  |
| Inferior parietal cortex (R) | 50 | -50 | 44 | 2.98 |  |  |
| ***AVH-*** |  |  |  |  |  |  |
| Precuneus (R) | 8 | -66 | 30 | 4.79 | 4157 | <0.001 |
| Precuneus (L) | -8 | -66 | 36 | 4.61 |  |  |
| Lateral ventricle atrium (L) | -30 | -48 | 8 | 5.55 | 465 | 0.009 |
| ***AVH+*** |  |  |  |  |  |  |
| Precuneus (L) | -10 | -66 | 34 | 4.98 | 3749 | <0.001 |
| Precuneus (R) | 10 | -66 | 42 | 4.74 |  |  |
| Dorsal anterior cingulate gyrus (L) | -2 | 30 | 22 | 4.76 | 670 | <0.001 |

L: Left; R: Right

**S5 Table. Activation during the Reversed condition (Reversed > Baseline).** For large clusters, regions encompassing the area of activation are indicated in brackets.

|  | **MNI coordinates** | | |  |  |  |
| --- | --- | --- | --- | --- | --- | --- |
|  | ***x*** | ***y*** | ***z*** | ***Z*** | ***k*** | ***p*** |
| ***Controls (HC)*** |  |  |  |  |  |  |
| Superior temporal Gyrus (L)  (Pre/post-central gyrus, IFG *triangularis*, IFG *orbitalis*, IFG *opercularis*, supramarginal gyrus, pallidum, putamen, thalamus, middle and inferior temporal gyrus, cerebellum, amygdala, hippocampus) | -46 | -18 | 4 | 9.95 | 36404 | <0.001 |
| Superior temporal gyrus (R) | 48 | -16 | 4 | 9.52 |  |  |
| Posterior superior temporal gyrus (L) | -34 | -30 | 12 | 9.39 |  |  |
| Inferior parietal gyrus (L) | -49 | -52 | 38 | 4.34 | 1041 | <0.001 |
| Supramarginal gyrus (L) | -54 | -38 | 50 | 4.04 |  |  |
| Inferior parietal (R) | 52 | -44 | 48 | 3.45 | 423 | 0.0152 |
| ***Patients (SZ)*** |  |  |  |  |  |  |
| Superior temporal gyrus (R)  (Pre/post-central gyrus, IFG *triangularis*, IFG *orbitalis*, IFG *opercularis*, supramarginal gyrus, pallidum, putamen, thalamus, middle and inferior temporal gyrus, cerebellum, amygdala, hippocampus) | 58 | -26 | 4 | 10.3 | 40564 | <0.001 |
| Superior temporal gyrus (L) | -48 | -18 | 4 | 10.2 |  |  |
| Postcentral gyrus (R) | 8 | -24 | 56 | 4.39 | 578 | 0.0020 |
| Supplementary motor area (R) | 4 | 2 | 66 | 4.58 | 527 | 0.0038 |
| ***AVH-*** |  |  |  |  |  |  |
| Superior temporal gyrus (R)  (IFG *orbitalis*, middle and inferior temporal gyrus) | 58 | -26 | 4 | 8.68 | 12302 | <0.001 |
| Superior temporal gyrus (L) | -48 | -18 | 4 | 8.27 | 8552 | <0.002 |
| Posterior superior temporal gyrus (L) | -48 | -26 | 6 | 7.95 |  |  |
| Cerebellum (L) | -16 | -78 | -34 | 4.47 | 2454 | <0.001 |
| Inferior occipital gyrus (L) | -26 | -88 | -16 | 3.91 |  |  |
| Fusiform gyrus (R) | 12 | -88 | -16 | 3.88 |  |  |
| IFG *triangularis* (L) | -56 | 18 | 26 | 4.73 | 1228 | <0.001 |
| Post-central gyrus (L) | -52 | -10 | 52 | 3.94 |  |  |
| IFG *opercularis* (L) | -48 | 18 | 18 | 3.9 |  |  |
| Middle frontal gyrus (L) | -34 | 6 | 38 | 3.6 |  |  |
| Hippocampus (L) | -28 | -10 | -22 | 5.33 | 778 | <0.001 |
| Cerebellum (R) | 16 | -80 | -42 | 5.08 | 406 | <0.001 |
| Thalamus (L) | -14 | -28 | -4 | 7.07 | 346 | <0.001 |
| ***AVH+*** |  |  |  |  |  |  |
| Superior temporal gyrus (R)  (Pre -central gyrus, IFG *triangularis*, IFG *orbitalis*, IFG *opercularis*, supramarginal gyrus, thalamus, middle and inferior temporal gyrus, amygdala, hippocampus) | 46 | -18 | 6 | 8.5 | 14255 | <0.001 |
| Posterior superior temporal gyrus (R) | 48 | -30 | 8 | 8.03 |  |  |
| Superior temporal gyrus (L)  (Supramarginal gyrus, middle and inferior temporal gyrus) | -48 | -18 | 2 | 8.66 | 8636 | <0.001 |
| Posterior superior temporal gyrus (L) | -36 | -30 | 10 | 8.48 |  |  |
| Cerebellum (L) | -14 | -80 | -40 | 6.23 | 3403 | <0.001 |
| Cerebellum (R) | 12 | -80 | -42 | 5.3 |  |  |
| IFG *opercularis* (L) | -38 | 8 | 26 | 4.58 | 1055 | <0.001 |
| IFG *orbitalis* (L) | -38 | 28 | -4 | 3.89 |  |  |
| Middle frontal gyrus (L) | -32 | 6 | 36 | 3.66 |  |  |
| IFG *triangularis* (L) | -42 | 28 | 6 | 3.41 |  |  |
| Post-central gyrus (L) | -4 | -40 | 68 | 3.54 | 583 | >0.001 |
| Pre-central gyrus (L) | -48 | -2 | 44 | 3.95 | 579 | >0.001 |

IFG: Inferior frontal gyrus; L: Left; R: Right

**S6 Table. Deactivations in the Reversed condition (Baseline > Reversed).**

|  | **MNI coordinates** | | |  |  |  |
| --- | --- | --- | --- | --- | --- | --- |
|  | ***x*** | ***y*** | ***z*** | ***Z*** | ***k*** | ***p*** |
| ***Controls (HC)*** |  |  |  |  |  |  |
| Precuneus (R) | 22 | -52 | 22 | 4.82 | 1570 | <0.001 |
| Fusiform gyrus (R) | 36 | -38 | -10 | 4.66 |  |  |
| Posterior thalamic radiation (R) | 36 | -48 | 2 | 4.25 |  |  |
| Fusiform gyrus (L) | -34 | -40 | -10 | 4.35 | 1465 | <0.001 |
| Superior occipital gyrus (L) | -16 | -68 | 40 | 3.67 |  |  |
| ***Patients (SZ)*** |  |  |  |  |  |  |
| Lateral ventricle/parahippocampal gyrus/Precuneus (L) | -32 | -44 | 0 | 5.7 | 5360 | <0.001 |
| Superior/middle frontal gyrus (L) | -26 | 32 | 36 | 3.64 | 418 | 0.016 |
| Superior/middle frontal gyrus (R) | 22 | 48 | 16 | 3.52 | 384 | 0.026 |
| Middle occipital gyrus (R) | 42 | -74 | 34 | 4.94 | 373 | 0.031 |
| ***AVH-*** |  |  |  |  |  |  |
| Lateral ventricle/parahippocampal gyrus (L) | -32 | -50 | 6 | 4.32 | 625 | <0.001 |
| ***AVH+*** |  |  |  |  |  |  |
| Precuneus (R) | 22 | -54 | 22 | 4.88 | 3045 | <0.001 |
| Precuneus (L) | -8 | -64 | 58 | 4.39 |  |  |
| Posterior thalamic radiation (L) | -36 | -48 | -2 | 4.29 | 392 | <0.023 |

L: Left; R: Right
